# Evidence of Members of the Royal College of Surgeons, before the British Parliament

**Published:** 1835

**Authors:** 


VII.—*Abstract of the Evidence of Members of the Royal College of Surgeons in London, taken before the Parliamentary Medical Committee, in* 1834.

We presume that many of our readers have for some time past seen occasional notices of the piracy committed by Sir Everard Home on the reputation and works of the celebrated John Hunter. It may not be improper to state here, that Sir Everard was the brother-in-law of Mr. Hunter, and as such was appointed, in 1793, in connection with Dr. Matthew Baillie, executor of the last will and testament of Mr. Hunter. It was in this way that his manuscripts, physiological and pathological, came into the possession of Sir Everard Home. And it was from these stupendous MS. volumes, comprising the labors of Mr. Hunter's life, that Sir Everard compiled those papers which were published in the Philosophical Transactions. They did indeed promise their *supposed author* an imperishable fame; but he has scarcely been numbered with the dead, till the irrefutable evidences of his guilt have been promulgated to the world. We subjoin the evidence of Mr. Clift, extracted from the London Lancet of July, 1835, for which we are indebted to the United States Medical and Surgical Journal. It is as follows:

### Evidence of Mr. W. Clift.

211. "What situation do you hold at the College of Surgeons?"

"I have been Conservator of the Museum since the year 1800, and was appointed by the College, the Hunterian collection being placed at that time at No. 13, Castle-street, Leicester-square, which communicated with the house that Mr. Hunter lived in, and which was the house in which his collection was originally deposited. The collection was removed thence to Lincoln's-inn-fields, where at first it was deposited in the dwelling house. When half the museum room was built, the collection was conveyed into that half, and there deposited until the remainder should be completed, which was done so far as to be open to visitors in 1813. The trustees of the museum recommended to Parliament in 1806, that 15,000*l.* should be granted towards building the college, on the council binding themselves to complete the buildings *within three years*, without any ulterior expense to Parliament, but when those three years had expired, the building yet *remaining incomplete*, Parliament, in 1810, voted 12,500*l.* more, when the council again undertook to make good any further deficiency out of the funds of the college. Thus from the removal of the collection in 1806, until the completion of the building in 1813, the collection remained quite inaccessible. It was in store-rooms, in fact,—in the dwelling-house, from the top to the bottom. While the collection remained in Castle-street, it was accessible to visitors, though but very few came. When I was first appointed conservator in 1800, the manuscripts of Mr. Hunter, which were descriptive of the collection in Castle-street, consisted of twenty-four fasciculi, relating to the physiological part of the collection; two volumes relating to the pathological series; and one volume relating to the fossils. The manuscripts descriptive of the pathological preparations, contained the history of very numerous cases relating to particular specimens, with such references that a good anatomist and physiologist would have been enabled to connect both preparation and manuscript; but the loss of these descriptions has greatly reduced the interest and value of the corresponding preparations; although when the government purchased the collection for the public, all the papers and manuscripts bearing any reference to that collection ought to have been given up with it, as public property; for Mr. Hunter, in his will, said, 'I also give to the said Matthew Baillie and Everard Home all my collection of natural history, and the cases, and other things belonging thereto, or used therewith, upon trust that they offer the same for sale in one entire lot to the government of Great Britain.' From the time of Mr. Hunter's death, on the 16th of October, 1793, and during nearly two years before that, (while I was Mr. Hunter's apprentice,) although not regularly appointed conservator, I had the entire charge of the collection. At the period of Mr. Hunter's death, and for some time afterwards, those manuscripts were deposited with the collection in an ante-room, and I clearly understood that they were to go along with the collection; but shortly before the collection was transferred to the College of Surgeons, those manuscripts were removed away from the collection, and I was first made aware that those manuscripts had been separated from the collection only when the collection was about to be delivered into the possession of the college by the executors of Mr. Hunter. Those manuscripts were then *taken by me in a cart to Sir Everard Home's house, by his order*, under the impression entertained by the trustess, that Sir Everard was the only person who could make a catalogue of the collection, but I had no conversation with Sir Everard at the time as to his removing them. He merely said that those papers, being a very large proportion of them loose fasciculi, were not fit for the public eye; and therefore he should take them into his keeping, for the purpose of using them in describing the collection. I did not make any remark upon that, and the trustees of the college were not made acquainted with the removal of the papers; nor was any inquiry made of me upon the subject."

212. "Would it have been possible for you, between the years 1806 and 1813, to have availed yourself of the manuscripts to compare them with the preparations?"

"It was almost impossible. The collection was put in order entirely under Sir Everard Home's direction, by me, but I had no access to the manuscripts which he had removed. Indeed, they were not necessary at that time."

213. "Why not?"

"Because the preparations were numbered, and we had merely to get them into their places according to the three folio catalogues which from 1794 down to 1806 had been prepared, two by myself, and a third by Dr. Shaw; but those three catalogues did not enter into the history of its preparations. A general arrangement of the collection on the shelves of the new museum was completed, *in a manner*, in 1813, and even if I could have had access to the manuscripts that had been removed, I was not in a condition to make use of them until 1817, and then it was expected that Sir Everard would prepare the descriptive part of them, I being only engaged under his direction, the catalogue being supposed to be the work of Sir Evarard. He merely employed me to transcribe whatever he pleased to direct. In 1817 he began to re-arrange the collection on the shelves, but he did not supply the description of each particular preparation as he proceeded."

214. "Did it ever occur to you that in order to make a complete description, it would be necessary to have recourse to the original manuscripts?"

"Without doubt, it frequently did. In fact those reflections suggested themselves to my mind when the manuscripts were first removed, but I did not communicate with the council or any individual members of the council respecting them, until 1823."

215. "Did Sir Everard Home repeatedly promise the board of curators to draw up a descriptive catalogue?"

"He was constantly urged, *quarterly*,* by the trustees to make progress in it."

216. "The council have stated, that in 1816 it was proposed that all the curators should become joint laborers in drawing up a descriptive catalogue, when Sir Everard Home, they say, declared that it was his special duty, and that he would admit of no participation in its performance: do you know any thing of that allegation?"

"Yes, every word of it is perfectly true; I heard him declare it. The trustees had been frequently urging the board of curators to make progress in the catalogue, and as they saw that no progress was making in it, they became anxious on the subject. This was in 1817, and was the cause of Sir Everard making the new arrangement of the preparations which I have mentioned, but he only produced a synopsis,—a very slight description of the general arrangement,—which was printed in 1818, but which did not at all enter into the particular history of each case; yet, as regarded the conservatorship of the museum and the board of curators, things remained on just the same footing as before, and I continued to receive my orders from Sir Everard Home alone. By-and-by, when I was appointed conservator, and began to prepare the catalogue, I expressed a wish to obtain some of Mr. Hunter's manuscripts; and then (in 1823) the board of curators learned for the first time the extent and nature of the manuscripts belonging to the collection left by Mr. Hunter. From 1817 to 1823 I had no access to any of the manuscripts which Sir Everard Home removed, except such as he brought with him to compare with specimens in the collection, for his own special purposes."

217. "What do you mean by *his own special purposes?*"

"When he was *preparing for his lectures*, or drawing up *papers for the Philosophical Transactions.*"

217*a*. "In giving him assistance in the arrangement, did you ever ask him to let you have access to the manuscripts?"

"No;† between 1800 and 1823 I never had any conversation with him

---

*Quarterly during nearly twenty years!

†Mr. Clift would seem deserving of blame for silence, or neglect, or torpidity, in this matter; yet who can doubt that had he been troublesome or inconvenient to the thief, he would have been removed from his office of assistant, and science would have been deprived of his services in rendering useful this invaluable collection? The instant that Mr.

on the subject of the manuscripts. During the whole of that period he was so intent on writing papers for the *Philosophical Transactions*, that *very little else, in the way of the collection, was thought of by him;** he *always* made his engagements in this way an excuse to the trustees for not having proceeded with the catalogue, saying that we were employed in making out the subjects which were but imperfectly understood. Though I believe that all the curators had but a very imperfect knowledge of the nature of those papers, yet *they knew that Sir Everard had in his possession all Mr. Hunter's manuscripts.* Besides which, almost all of them had been acquainted with Mr. Hunter, and *must* have known of the existence of a *large mass of manuscripts.* After the destruction of the papers, Mr. Cline stated to me, on Mr. Hunter's own authority, that Mr. Hunter had written a Treatise on Diseases of the Bones, which he intended for publication. Drawings intended to illustrate this treatise, and made by an artist who lived in his house, Mr. W. Bell, are preserved in the museum."

218. "When was it that you first received any information as to the destruction of those manuscripts?"

"I think in July, 1823. I first obtained a knowledge of the circumstance from Sir Everard himself. He began by telling me, that on that very week his house had been nearly on fire; that the engines came, and the firemen insisted upon entrance, as they saw the flames coming out of the chimney. He did not wish to admit them, but they insisted upon being admitted, and then he told me that it was in burning those manuscripts of Mr. Hunter that the fire occurred. This conversation passed in a chaise on our road to Kew, where was a monthly meeting called the 'medico-botanical.' I can hardly describe my feelings on receiving this information. I said to him, 'I hope, Sir Everard, you have not destroyed those ten volumes relating to the gallery and Mr. Hunter's lectures?' he replied that he had; and then I mentioned to him perhaps twenty others, of which I had a very perfect recollection (and I felt that all the hopes which I had entertained were entirely frustrated and destroyed; my life had been spent in the service of that collection, and I hoped to have lived to see those papers beneficially employed;) but he told me that they were *all gone*, and then said I to him, 'Well, Sir Everard, there is only one thing more to do, and that is, to burn the collection itself.' We had very little conversation after that for a very considerable time. I considered that an irreparable injury had been done to the collection. That week Sir Everard had received back from the printer the last proof of his second volume of Lectures on Comparative Anatomy; and I knew *that he had used those papers very largely in the composition of that work.* Four or five days afterwards I made a communication on the subject to the members of the council, first communicating it to the chairman of the board of curators, Sir William Blizard, in private. A board was summoned very quickly in consequence of that, and I was called before it, but Sir Everard was not. Having learned during my conversation with Sir Everard that he had not destroyed all the manuscripts relating to the pathological prepara-

---

Clift became wholly independent of the thief, the board of curators, that worse than incapable body, were made acquainted with the want which led to the discovery of the robbery.

*Yet all this time the farce of urging the thief to "draw up a descriptive catalogue," underwent a quarterly performance by the trustees of the museum!

tions, for (he had said, 'I have some of those') at this board of curators I mentioned that circumstance, telling them that some were according to his own confession still in existence, and they of course did not need my advice to apply to Sir Everard for them."

219. "Will you refer to any memoranda you have on the subject, and state, as nearly as you can, what the papers were that were destroyed?"

"Among them were nine folio volumes of dissections of animals, viz: Vol. I. Ruminants Vol. II. Animals *sine cœco*. Vol. III. Monkey and its Gradations. Vol. IV. Lion and its Gradations. Vol. V. Scalpris Dentata. Vol. VI. Anatomy of Birds. Vol. VII. The Tricolia. Vol. VIII. Anatomy of Fishes. Vol. IX. Anatomy of Insects. There was one volume on the Natural History of Vegetables. There was also a great number of fasciculi, among which were the following:— Introduction to Natural History; numerous Physiological Observations; Comparative Physiology; Comparison between Man and the Monkey; On Muscular Motion, being subjects of Croonian Lectures; Effects of Extracting one Ovarium upon the number of young produced; Experiments on Ewes, with a view to determine Impregnation and Uterine Gestation; On Monsters; On the Skeleton; Dissection of the Tapir; Dissection of the Armadillo with nine bands; Animals from New Holland; Piked Whale, Bottle-nosed Whale, Fin-backed Whale, Porpoise; Worms in Animals of the Whale Tribe; Bell-Barnacle; On the Eel; Anatomy of the Holothuria; Anatomy of the Siren of North America; Account of the Unicorn Fish from Hispaniola; The Earth Worm; Progress and Peculiarities of the Chick; Description of Rymsdyk's Drawings of the Incubation of the Egg; General Observations on Insects, the Bee Tribe, Humble-Bee, Wasp, Hornet, and Beetles; Anatomy of the Silk-Worm; Anatomy of the Moth; Red-piped Coral. On Fossil Bones, two parts; Observations on Surgery; Observations on Scrofula and Cancer; Lectures on Principles of Surgery; Cases with *post-mortem* examinations; Cases where no *post-mortem* examinations were obtained; Two cylinders of Cases, written out separately and fairly. Two volumes in folio were recovered by the curators on application to Sir Everard, perhaps a tenth part of the whole of the papers relating to the preparations in the collection."

220. "Do you find any thing in reading Sir Everard Home's Lectures that in any way *supplies the loss* as regards the descriprion of preparations in the museum?"

"Sir Everard's is a mere general description; it seldom refers to particular specimens."

221. "Were you old enough, at the time of Mr. Hunter's death, to have any conversation with him on the subject of these papers, so as to know whether he attached any value to them?"

"I was Mr. Hunter's apprentice from February, 1792, till his death, on the 16th of October, 1793. During that period I was employed every evening in writing for him, and many of those manuscripts were in my own handwriting. I had no conversation with him on the subject, of course, because I was but a boy, and had merely to transcribe what he gave me to transcribe, in the same room with him. I was employed in taking care of the collection all day long, and frequently transcribed portions which were in loose notes, into volumes which he would take down for that purpose."

222. "Did you ever hear any hint from him of his wish that those papers should be destroyed?"

"Never; nor did I ever hear, either from Sir Everard Home or from Dr. Baillie, his co-executor, any hint whatever of any directions that Mr. Hunter had given for the destruction of the manuscripts."

223. "Will any industry that you or your fellow-laborers at the museum can bestow upon it, ever repair the loss which it has sustained by the burning of those manuscripts?"

"No, it is impossible. Some of the preparations have been rendered wholly useless by the loss of the accompanying description. It is very frequently necessary to re-examine the organs of animals that are daily presenting themselves to us, in order to throw light upon the specimens in the museum, and to prove what they really are; because many of them (the descriptions being absent) are utterly unknown. The nature of many of them has been discovered, as it were, by these means. We preserve the unknown preparations, and endeavor to discover by degrees what they are. There may be, of all kinds *a thousand*, of wet and dry, yet remaining, that are unexplained. The loss is irreparable in the department of comparative anatomy."

224. "When the trustees wrote to Sir Everard Home, did they require an account from him of the papers he had destroyed?"

"No, not then, but they did so on receiving his answer, saying, *that he had destroyed them entirely*, that having used them for the purposes of the collection for so many years, and, AS A WINDING-UP OF HIS EXECUTORSHIP, he had, according to a promise he had made to Mr. Hunter, destroyed them. The letter was so worded that you would have taken it for granted that they were *all* destroyed. This letter was read to me by the trustees, and I said, 'Sir Everard told me that he had not destroyed them *all;*' and therefore that they ought to write to him to ask for those that had not been destroyed. They said, '*How can we do so?* because his letter expressly states that they are all destroyed.' To this I replied, 'Then Sir Everard must have told me an untruth,' and then I told them what parts he had assured me were *not* destroyed. They wrote to him, accordingly, for those papers, AND HE SENT THEM *such as he pleased!* A very small parcel, in the first instance, was sent by Sir Everard to the trustees. Those papers were put into my hands, to see how far they were applicable to the purposes of describing the collection. Some of them referred to others that *I knew did exist*, but were only a very small proportion of those that I had a perfect recollection of. A second application was then made for more, and that extracted a much larger parcel. I am not sure whether there was any further application."

225. "Were any applications made to *his executors*, to inquire whether there were any remaining papers of Mr. Hunter in their possession?"

"Yes, with considerable success. The volume that was mentioned, respecting vegetables, referring to a part of the collection, from which I have been able to explain some of the vegetable preparations in a way that we could not have done without them, was recovered; and there were some that were possibly Sir Everard Home's, that also threw light upon many of the preparations. These were *duplicates of some of the papers of Mr. Hunter*, yet at the time of the last recovery of papers form Sir Everard Home himself, he stated *that those were all the papers he had retained in his possession.*"

226. "Do you conceive that if Mr. Hunter had given him directions to destroy his papers after they had been made available for the purposes of the collection, Sir Everard really had *complied* with those directions, and had extracted from them all the valuable information they contained relating to the collection, and applied it to illustrate the preparations in the museum?"

"Certainly not."

227. "Therefore, according to his own statement, he had destroyed them *before he had complied with the directions of the testator;* if, indeed, those directions were ever given?"

"*Certainly.*"

228. "Did Sir Everard Home ever state where and when these directions were given by Mr. Hunter?"

"I had heard it stated, but I do not know by whom, that it was when Mr. Hunter was dying, *which I knew could not be true*, because I was the last person in his family who saw him alive, and I knew that Sir Everard *was not present at his death.* This I told Sir Everard at the college, five or six months after the conversation which took place on our way to Kew, and I told him so in consequence of what had passed between him and the trustees."

229. "Was it in the presence of others?"

"No, it was alone, it was a very rambling conversation, an hour and a half long, I believe."

230. "Did Sir Everard begin the conversation with you, or did you begin with Sir Everard?"

"He came to the collection, and it was the only conversation I ever had with him after the first conversation on the destruction of the papers. It was entirely on the subject of those papers. He told me, that I must have known that the papers were not fit to be submitted to the public eye, because they were in such a state. He said the spelling was not very correct. I said, that he knew very well that if this catalogue ever was to be made, the *labor* of it must come to my share, and that I did not consider my eye to be the public eye; and I added, that he would have been welcome to all the credit of the catalogue, so that the thing had been but done: but he said that directions were given to him by Mr. Hunter to destroy the manuscripts when he was dying; and I said, 'That is impossible.'"

231. "What was his remark upon that?"

"He made no answer."

232. "Did he leave the impression with you that such a direction had been, or that it had not been given?"

"If he had sworn it, I would not have believed it. The duration of Mr. Hunter's illness was not a minute. He died of a disease of the heart."

233. "Were you employed by Sir Everard in making drawings for his papers in the *Philosophical Transactions*, or in other publications?"

"All my life, from the time that I was first acquainted with him, till the time of this unfortunate occurrence,—that is, till the time of the conversation on the way to Kew."

234. "Did that conversation entirely interrupt the friendship that you before had with Sir Everard?"

"Entirely; I had no other cause for its interruption."

235. "Why do you suppose that the manuscripts of Mr. Hunter were used by Sir Everard in preparing papers (for the Philosophical Transactions or other publications) bearing his name?"

"*Because I frequently transcribed parts of Mr. Hunter's original*

*papers and drawings into the papers which were to appear in Sir Everard's name.*"

236. "When he made his first avowal of the destruction of the manuscripts, did he appear to have made it involuntarily, or how did it happen?"

"It was, I believe, to see how I would take it; but though there had been a regular application to him *every quarter* to complete the descriptive catalogue, yet as I said before, the papers were not destroyed until immediately after receiving the last proof sheet of his second volume."

237. "*Was any meeting held for censuring Sir Everard Home, or for excluding him from the council?*"

"Not to the best of my knowledge."

238. "Did he continue to be a member of the council until near the day of his death?"

"I am not sure. He died a trustee of the museum."

239. "Is there any thing further relating to this deplorable transaction that you wish to state to the committee?"

"I do not recollect any thing further. I do not know that the council of the college ever came to any resolution on the subject of Sir Everard's conduct, nor have I ever heard that the nature of the loss was communicated to the government by the trustees. The destruction of the papers happened immediately previous to the death of Sir Everard Home's co-executor, Dr. Baillie, perhaps a month or two, but he was so ill that it was impossible that he could have interfered, even had he known of the circumstances. He might have been considered as not acting in the business; because Sir Everard very rarely communicated with him. Sir Everard was Mrs. Hunter's brother, and took all the active part. No communication was made to Dr. Baillie on the subject previous to his death."

240. "Has not the want of access to the manuscripts descriptive of the preparations, very greatly added to the time and labor required to describe the collection?"

"Most unquestionably it has, in a very material degree. Moreover I felt that if I could describe the collection in Mr. Hunter's own words, the public would be better pleased with it, whether Mr. Hunter was right or wrong. It might have been altered or improved by any one who thought himself competent to do so; as it is, we are obliged to trust to our own resources and knowledge, instead of that of Mr. Hunter."

241. "How was it that you became so intimately acquainted with the nature of Mr. Hunter's manuscripts that were destroyed?"

"After Mr. Hunter's death till 1806, when the collection was transferred to the college of Surgeons, I had the key of the cases which contained them, and was anxious to make myself acquainted with the nature of their contents, as they related chiefly to the preparations. I had also, I may say, no other books to read at that time; so I frequently availed myself of the opportunity to read them, and make extracts from them, and I know that they related, many of them, entirely to the preparations in the museum. Thinking there was a great deal of useful information in them, I made large extracts from some of them, and have been instrumental in preserving, in substance, by means of those extracts, I hope nearly half."

242. "Did Sir Everard Home ever make to the curators or trustees of the museum any denial of the statements you made to them as to the number and contents of the manuscripts?"

"No, not a single denial."

243. "Up to the destruction of the manuscripts, on what terms of intimacy were you with Sir Everard?"

"I looked upon him as one of my oldest and best friends; to that hour we had never had the slightest disagreement, but this unfortunate event put a stop to that intimacy: for I considered the obligation which I owed to the memory of Mr. Hunter, to the trustees, and to the curators, superior to any private obligation which I might have been under to Sir Everard Home, and that was my only object in mentioning the subject, which I wished it had never been necessary for me to disclose."

It is a matter of deep regret, that one who was calculated to adorn science, and shed a lustre on the profession of which he was a member, should have been so destitute of self respect, as to violate a trust, than which none more important could have been committed to his hands,—the property, reputation and honor of a deceased brother.

The inquiry has been raised—Why was this disclosure not made long since, and during the lifetime of Sir Everard? We reply that, according to the testimony of Mr. Clift, it was made to the trustees of the college as early as 1823, but there is no evidence of their having made any report to the government on the subject. The matter was permitted to sleep in silence till the parliamentary committee instituted their inquiries, by which the conduct of Sir Everard was brought before the gaze of the scientific world. The dishonorable employment of the manuscripts of John Hunter for purposes of self-aggrandizement, will consign the name and the memory of Sir Everard Home to merited obloquy and contempt.

